# Physical Forces and Transient Nuclear Envelope Rupture during Metastasis: The Key for Success?

**DOI:** 10.3390/cancers14010083

**Published:** 2021-12-24

**Authors:** Benoit R. Gauthier, Petra I. Lorenzo, Valentine Comaills

**Affiliations:** 1 Andalusian Center for Molecular Biology and Regenerative Medicine-CABIMER, Junta de Andalucía-University of Pablo de Olavide-University of Seville-CSIC, 41092 Seville, Spain; petra.lorenzo@cabimer.es; 2 Centro de Investigación Biomédica en Red de Diabetes y Enfermedades Metabólicas Asociadas (CIBERDEM), 28029 Madrid, Spain

**Keywords:** metastasis, circulating tumor cells, mechanostress, nuclear envelope rupture, cGAS/STING, chromosomal instability, EMT, SASP

## Abstract

**Simple Summary:**

Metastasis is the process that allows the seeding of tumor cells in a new organ. The migration and invasion of cancer cells involves the pulling, pushing, and squeezing of cells through narrow spaces and pores. Tumor cells need to cross several physical barriers, such as layers of basement membranes as well as the endothelium wall during the way in and out of the blood stream, to reach the new organ. The aim of this review is to highlight the role of physical compression in the success of metastasis. We will especially focus on nuclear squeezing and nuclear envelope rupture and explain how they can actively participate in the creation of genomic heterogeneity as well as supporting metastasis growth.

**Abstract:**

During metastasis, invading tumor cells and circulating tumor cells (CTC) face multiple mechanical challenges during migration through narrow pores and cell squeezing. However, little is known on the importance and consequences of mechanical stress for tumor progression and success in invading a new organ. Recently, several studies have shown that cell constriction can lead to nuclear envelope rupture (NER) during interphase. This loss of proper nuclear compartmentalization has a profound effect on the genome, being a key driver for the genome evolution needed for tumor progression. More than just being a source of genomic alterations, the transient nuclear envelope collapse can also support metastatic growth by several mechanisms involving the innate immune response cGAS/STING pathway. In this review we will describe the importance of the underestimated role of cellular squeezing in the progression of tumorigenesis. We will describe the complexity and difficulty for tumor cells to reach the metastatic site, detail the genomic aberration diversity due to NER, and highlight the importance of the activation of the innate immune pathway on cell survival. Cellular adaptation and nuclear deformation can be the key to the metastasis success in many unsuspected aspects.

## 1. Mechanical Stress during Metastasis

Metastasis is the process by which tumor cells spread from a primary tumor site to a distant location. In order to succeed, cancer cells need to invade the basement membrane, migrate into the connective tissue, enter into the blood stream or the lymphatic system, survive in the circulation, exit from the circulation, and finally colonize secondary tumor sites.

### 1.1. Mechanics behind Metastasis

In order to seed and create new metastasis in invading organs, tumor cells must travel in and out of the blood stream, defying several mechanical cues during their journey.

#### 1.1.1. Migration through the Basement Membrane

**Figure 1:**
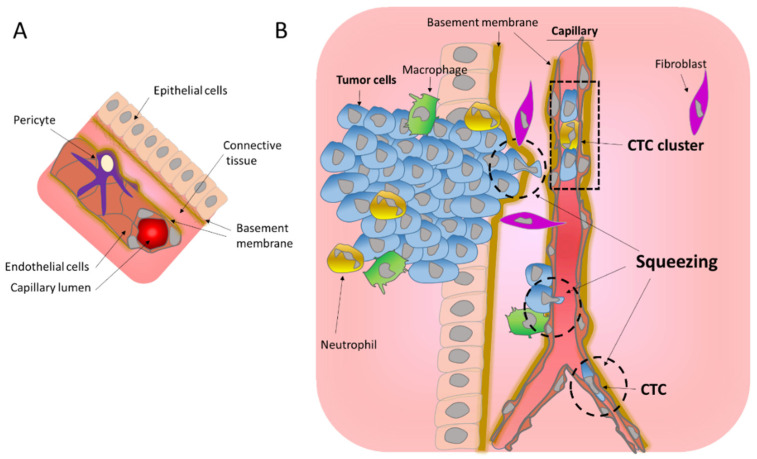
Mechanical challenges affecting tumor cells during migration. (**A**) Schematic representation of a normal blood vessel within its environment. (**B**) Intravasation of tumors cells into the circulation. Invading tumor cells cross the basement membrane and migrate through the connective tissue to reach the endothelial wall, which they cross to enter in the blood stream. All these steps involve important squeezing of the cells and their nuclei (hatched circle) imposing mechanical challenges. Tumor cells in the blood stream can be found as single Circulating Tumor Cells (CTC) or as CTC clusters.

During the metastasis, cancer cells need to cross the basement membrane (BM) several times, which is a thin layer of the Extra Cellular Matrix (ECM) that separates both the epithelial and endothelial cells from the underlying tissue, and represents a structural barrier to tumor cell migration and invasion (Figure 1) [1]. The BM is a dense nanoporous sheet, with pore sizes between 10 and 112 nm, shaped by two major ECM macromolecule proteins, collagen IV and laminin, self-assembled into two supramolecular polymers [1,2]. The mechanisms underlying cancer cell invasion of the BM are still not completely understood. Several studies have established the importance of proteases-mediated degradation of the BM during invasion [3,4,5], notwithstanding, protease-independent mechanisms have also been implicated [6]. In the latter, the invading tumor cells can form invadopodia protrusions to mechanically open up micron-sized channels in the matrix to then squeeze and migrate through them [7,8,9]. This migration can be of single cells or of groups of cells that then form CTC clusters within the blood stream [10]. Additionally, it has been suggested that Cancer-Associated Fibroblasts (CAF), a group of activated fibroblasts, can guide the migration of cancer cells. CAF can pull, stretch, and soften the BM, leading to the formation of gaps through which cancer cells can migrate [11,12,13].

#### 1.1.2. Transendothelial Migration

Once they have crossed the BM, cancer cells need to enter and exit blood vessels to colonize secondary tumor sites. In order to shuttle in (denoted as intravasation) and out (denoted as extravasation) of the blood stream, invading tumor cells need to cross the endothelial walls of blood vessels and as such are confronted by several mechanical challenges. These steps rely on the intrinsic properties of cancer cells such as the epigenetic state, the composition of the microenvironment, and the mechanical cues associated with this process (reviewed in [7]).

Blood vessels formed by endothelial cells organized into a tube and wrapped by pericyte cells (Figure 1A), can be very different in size ranging from 25 mm in diameter in the case of the aorta to as few as 5 to 10 μm in the case of the capillaries. As opposed to large blood vessels, capillaries are formed by a single layer of the endothelium allowing the exchange of nutrients mainly through diffusion. It is noteworthy that tumor blood vessels are morphologically different from normal blood vessels. They are characterized as tortuous and leaky, with abnormal basement membranes and with abnormal pericytes [14]. Pericytes on tumor capillaries are loosely associated with endothelial cells and exhibit an abnormal shape, sometimes extending their processes away from the endothelium and towards the tumor [15]. These tumor capillary abnormalities might facilitate the entrance of tumor cells into the blood stream.

To enter in and exit from the blood stream, cells need to cross the endothelial walls. In the better studied case of leukocyte transendothelial migration, it has been demonstrated that this involves breaching the endothelial cell–cell junctions to allow the transmigration of leukocytes across the endothelial walls [16,17,18]. The extravasation is completed by rolling and crawling on the endothelium to adhere and probe endothelial cells [16,17,18]. When attached to it, leukocytes develop protrusions through adjacent endothelial cells, leading to transendothelial migration [16]. Importantly, this constricted migration imposes extreme nuclear shape changes that involve the formation and insertion of a nuclear lobe and nuclear squeezing [19]. In a similar way during intravasation, invading tumor cells generate protrusions that are initially aligned along endothelial cell–cell junctions and are then inserted between the endothelial cells to disrupt their connection allowing transendothelial migration [7,8,9,20]. The presence of macrophages in the proximity of the intravasation sites suggests that these immune cells may play a role in the opening of the endothelial wall [9,13,20]. It is noteworthy that the intravasation of tumor cells can occur as single cells as well as groups of cells that form CTC clusters, ranging from 2 to 50 cells [21,22]. These CTC clusters can travel in the blood stream and are associated with neutrophils [22,23] and/or CAF [11]. Inside the capillaries, CTC clusters can align into a single lane without disturbing their tight junction and remaining attached [24]. Concerning the exit, whether the extravasation of CTC involves rolling and crawling for attachment to the endothelium as observed for leukocytes is still unknown. However, since CTC extravasation mainly occurs at the capillaries, the constriction derived from the capillary size will likely favor the attachment of the CTC to the endothelium (Figure 1B) [9]. Additionally, tumor cells are often aneuploid with a history of whole genome doubling, hence their larger size [25], and thus are more likely to become trapped in the capillaries, favoring their attachment to the endothelium.

Importantly, cell migration during intravasation and extravasation entails squeezing, and mechanical challenges derived from crossing through narrow spaces up to only 5 microns in the capillaries and even smaller in BM pores (Figure 1B). Because cells are highly dynamic and adaptive, such movements are possible. However, stretching, and physical pressure can have profound effects on cellular reprograming through mechanosensitive signaling and survival, and thus they affect tumor progression.

### 1.2. Cellular Plasticity and Mechanosensitivity under Migration

Cell motility is driven by the cytoskeleton through the generation of traction forces that cause drastic cytoplasmic changes allowing several kinds of cell motion. Migrating cells typically adopt a polarized morphology with a leading edge containing a protrusive branched F-actin network, and a trailing edge containing a contractile actin–myosin network [26]. Both the cell biology and traction forces generated by the cytoskeleton have been studied extensively in past decades, leading to a fairly complete description of their behavior and regulation during migration [26]. However, much less is known on the role of the nucleus and its dynamic ability to adapt and protect the genome during migration.

The nucleus is compartmentalized from the cytoplasm by the Nuclear Envelope (NE), a lipid bilayer reinforced on its inner side by the nuclear lamina, a sheet-like structure of diverse proteins such as Lamin A and B as well as other components (For a review see [27]). The nucleus is generally rounded and stiffer than the cytoplasm [28], and thus can be the rate limiting step to the constriction imposed by cell migration. In motile cells, the nucleus undergoes controlled rotation, promoting its alignment with the axis of migration and favoring a better positioning through constricted areas [29,30]. Furthermore, upon the modulation of several NE proteins such as lamins A and B and their ratio, the nucleus can acquire viscoelastic properties that allow its adaptation to constriction [31,32]. A special case are the neutrophils that circulate in the blood and need to infiltrate tissues and challenge mechanical constrictions. These cells harbor a multi-lobed and highly flexible nucleus that allows them a 100 times faster migration than fibroblast or cancer cells [33]. Interestingly, cancer cells that enter the epigenetic program Epithelial to Mesenchymal Transition (EMT) that confers migration and invasiveness properties, gain nuclear flexibility and nuclear deformability by decreasing their amount of nuclear envelope proteins such as lamins A and B [34] to adapt to future squeezing needs. Interestingly, this downregulation has been observed at the protein level while an upregulation at the RNA level has been detected, making their study challenging in patient-derived samples [34].

**Figure 2:**
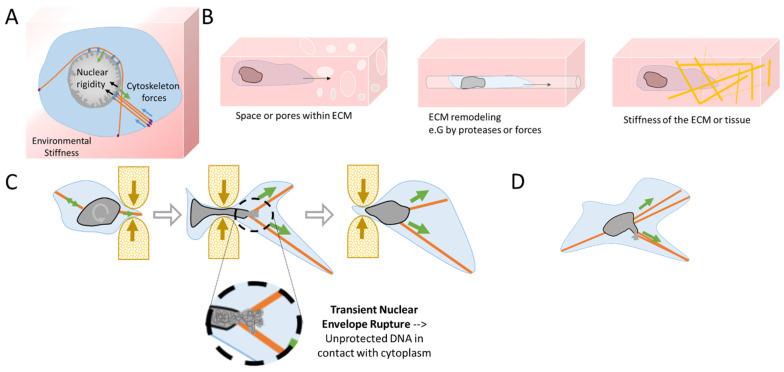
Mechanical challenges during cell migration lead to transient Nuclear Envelope Rupture (NER). (**A**) Homeostasis in physical forces from the environmental stiffness of the Extra-Cellular Matrix (ECM) (blue arrows), the cytoskeleton (green arrows), and the nuclear rigidity (black arrows—chromatin rigidity and NE composition). At focal adhesions (violet ovals) the mechanical forces are transferred from the ECM to the cytoskeleton, which then transfers them to the nucleus via the LINC complex (blue ovals). (**B**) During migration, cells can confront diverse ECM structures such as narrow spaces and pores, channels generated by protease action, or by physical forces from the invadopodia from the previous passage of other cells, chemoattractant gradients, and different densities of specific fibers (Figure adapted from Yamada K.M. et al. [41]). (**C**) A cell passing through a constriction will rotate and position its nucleus to favor the required nuclear deformation. Cytoskeleton forces and extreme nuclear curvation during passage in a constricted area can lead to a transient NER, disrupting the proper nuclear compartmentalization. Unprotected DNA is then in contact with the cytoplasm until NE is repaired, restoring proper nuclear compartmentalization. (**D**) During cell migration, the cytoskeleton might also apply excessive forces to the nucleus, leading to blebbing and transient NER.

Cells are in constant homeostasis between the stiffness of the environment, the forces of the cytoskeleton, and the nuclear rigidity (Figure 2A). All are connected and sense each other to adapt and preserve their integrity. Cells are connected to the ECM through integrins located at the plasmatic membrane that contact with and transfer the tension to the cytoskeleton at the focal adhesions (FA). The cytoskeleton connects the plasmatic membrane with the NE via the linker LINC complex [35]. In order to adapt to new environments and to biophysical stress, cells have mechanosensitivity and mechanoresponsive mechanisms that allow them to rapidly adapt to their new location as tissues harbor different stiffness and can be soft as the brain, bone marrow, and fat, which involve little mechanical stress, or can be as stiff as muscle, cartilage, and bone, which sustain high levels of biophysical stress [31].

One of the most characterized mechanosensitive pathways is the Hippo pathway and its components YAP and TAZ that interact with FA [36,37]. Another mechanosensitive sensor and transducer of mechanical stress is the calcium-permeable Piezo channel [38], located at the plasmatic membrane, which can assist the cell in choosing the migration pattern under a certain level of pressure and can shift movements using blebs rather than pseudopods [39]. The matrix stiffness can also lead to several cellular changes such as the adaptation of the nuclear flexibility by the regulation of Lamin A degradation by phosphorylation [31] as well as by the remodeling of the cytoskeleton and modifications in cell adhesion [37,39]. Importantly, matrix rigidity also controls EMT via the mechanoresponsive EPHA2–LYN complex [40]. Thus, the environment stiffness can modulate and stimulate the migration and invasion of the cells upon certain conditions.

Interestingly, the shape of the nucleus is also mechanosensitive and mechanoresponsive. The stiffness, elasticity, and the presence of pre-existing pores or passageways influence the local ECM and how easily cells will migrate and will be able to pull, push, and squeeze their nucleus in it [41] (Figure 2B). Cells can either use mechanical forces or proteases to expand pores. In fact, migrating cells use their nuclei as a mechanical gauge to ‘measure’ the diameter of the passages through the ECM and to sense the path of least resistance [41,42]. However, when cells need to pass through constrictive areas, the nucleus can sense a limit point in its flexibility and curvation and triggers the loosening of chromatin compaction to facilitate the nuclear passage [43]. At this particular constriction threshold, the NE can also induce calcium release that activates myosin II to accelerate the movement for a faster rescue of the nucleus from the constraint area [44,45]. During the migration and passage through constrictions, the cytoskeleton imposes considerable traction forces, resulting in extreme nuclear deformation (Figure 2C,D) that can surpass the adaptation capacity of the NE. The resulting shape distortion can lead to NE blebbing and extreme curvation causing the collapse of the NE during the interphase [27]. The use of time-lapse imaging [34,46,47] has allowed the observation and quantification of the NE collapse, also called Nuclear Envelope Rupture (NER). This NE collapse can be a transient event, repaired by the ESCRT III machinery [47,48], or can be irreversible as in the case of micronuclei [48,49]. Notwithstanding, transient NER can last from a few minutes to up to one hour [34,46,47] with unknown consequences for genome integrity. Importantly, NER has been associated with cancer. Recent studies from Nader and coworkers showed the increase in NER at the invasive foci of human breast cancer and colorectal adenocarcinoma [50]. Moreover, NER has also been observed in vivo in invasive cells and CTC in mice models [46,47,50].

## 2. Consequences of Nuclear Envelope Rupture on Tumor Genomic Heterogeneity

### 2.1. Role of NER in Simple and Complex Chromosomal Rearrangements

**Figure 3:**
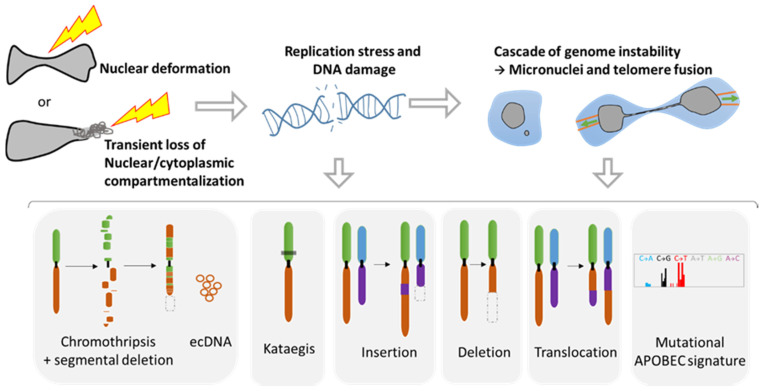
Genomic instability associated with nuclear squeezing and nuclear envelope rupture leads to the generation of genomic diversity. Nuclear deformation and nuclear envelope collapse, displaying a transient loss of compartmentalization, lead to replication stress and DNA damage. This DNA damage can initiate a cascade of genomic alterations in the next mitosis with the generation of micronuclei and telomere fusion, provoking a chromatin bridge. Such events are known to initiate a myriad of diverse genomic events such as chromothripsis, extra chromosomal DNA (ecDNA), the hypermutated pattern Kataegis, events of insertions, deletion, and translocation, as well as the mutational signature APOBEC (Adapted from Gauthier BR, et al. [27]).

The loss of proper nuclear compartmentalization can have drastic consequences in terms of genome evolution. During NER, the unprotected DNA in contact with the cytoplasm can result in DNA breaks [46,47,50,51] (Figure 3). Indeed, the use of DNA damage markers such as γH2AX and 53BP1 foci revealed important double strand DNA (ds-DNA) breaks in the constricted nuclei during migration through narrow areas [46,47,50,51,52]. Importantly, nuclear compression and deformation, even in the absence of NER, leads to increased replication stress in the S/G2 phase and provokes DNA breaks [51]. Moreover, in an elegant study, Nader and collaborators demonstrated that during NER, TREX1, a cytoplasmic DNA exonuclease that clears normal endogenous cytosolic DNA, can enter and become trapped inside the nucleus after NER repair causing massive DNA breaks [50]. Thus, the DNA damage observed during nuclear squeezing and NER can have profound consequences on tumor evolution.

Taking advantage of advances in high throughput sequencing and in single cell analysis technologies, a plethora of genomic anomalies have been discovered in cancer cells. Besides the classical and simple genomic aberrations such as mutations, deletions, translocations, and insertions, cancer cells can present complex chromosomal rearrangements such as chromothripsis and kataegis (Figure 3). Chromothripsis is a chromosomal instability phenomenon where hundreds of chromosomal rearrangements occur during one single event in a localized region of one or few chromosomes. This type of chromosomal rearrangement is highly frequent in cancer with a prevalence of 49% to up to 80% [53]. Chromothripsis is associated with the formation of circular extrachromosomal DNA (ecDNA) [54,55] as well as with segmental deletion. Kataegis is a pattern of localized hypermutations occurring in a small region of DNA.

Importantly, all these complex and simple chromosomic events are known to originate from the NER of micronuclei [55,56,57,58] or during the NER of cells presenting a chromatin bridge during a telomere crisis [59,60,61] (Figure 3). Micronuclei are small nuclei found next to the main nucleus in cancer cells that contain a full chromosome or a chromosome fragment, and are the result of aberrant mitosis [27]. It is noteworthy that the NE of these micronuclei is fragile and tends to disrupt without the possibility of proper repair [48,49]. On the other hand, the chromatin bridge appears when cells experiencing telomere fusion connect two daughter cells. This implies the generation of additional tension forces affecting the NE during movement, leading to NER that can last up to two minutes [60].

The mechanisms responsible for the genomic aberrations linked with NER are still under debate. Some authors have demonstrated that in the cytoplasm the unprotected DNA can be attacked by DNAses such as TREX1 or by the immune DNA mutator APOBEC upon NER [61]. The recent discovery of the nuclear internalization of TREX1 after NER repair reinforces its role in generating DNA damage [50]. APOBEC that plays a role against retrovirus attack can lead to the Kataegis pattern [61] or to the mutational signature APOBEC, characterized by an increase in mutations with the substitutions C-to-G and C-to-T [62]. Additionally, it has been demonstrated that during the chromatin bridge, the mechanical forces generated can trigger the breakage of the chromosome bridge, leading to extensive DNA breaks [58]. Furthermore, the loss of compartmentalization can affect the replication inside the micronuclei, provoking a desynchronization with the main nucleus. Thus, the main nucleus may start the mitosis too early for the DNA trapped inside the micronuclei, which is not folded nor protected and can lead to its pulverization within the cytoplasm, resulting in chromothripsis [56,58].

Altogether, these data reveal the profound and hereditary consequences that NER can have on the genome and the creation of genomic diversity. It is not yet known if the NER observed during migration [34] and passage through tight spaces [46,47] can also generate drastic complex genomic reorganization. Nevertheless, a recent study using CRISPR-Cas9 gene editing has shown that a single ds-DNA break can lead to a cascade of events resulting in the formation of micronuclei and chromosome bridges [63]. As such the DNA damage caused by nuclear deformation and NER can be amplified into far more extensive genomic alterations during subsequent mitosis (Figure 3), leading to a myriad of genomic diversity. Thus, the biophysics behind nuclear squeezing may be a major player in the increase in genomic heterogeneity during metastasis.

### 2.2. Metastasis and Genomic Evolution

**Figure 4:**
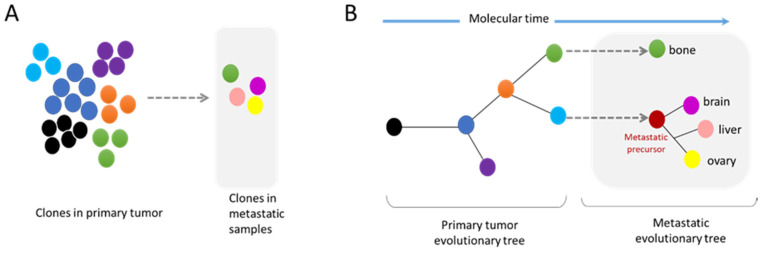
Modes of metastatic dissemination from the primary tumor. (**A**) The primary tumor is composed of a multiclonal population with cells competing between each other. Metastatic tumors are formed by clones found in the primary site, as well as by new independent subclones. (**B**) The phylogenetic tree shows the history of the tumor evolution. Genomic diversity can arise at all steps of tumor progression. Importantly, the metastatic site can be composed of clonal populations found in the primary tumor site or independent subclones.

In the past decade, extensive sequencing of numerous patient tumors revealed that cancer genomes are highly complex and heterogeneous. Tumors are composed of several clones with different genomic alterations that compete with each other. Upon specific conditions such as therapy treatment, some of these clones will be favored, thus becoming more prevalent than others (Figure 4A). In order to identify the major driver events for tumor initiation, the genome of tumor samples of the same patient but from different body locations and at different time points were sequenced to generate a phylogenetic tree that highlights the tumor history. The analysis of these phylogenetic trees revealed that complex structural events, including chromothripsis, are major drivers during the early phase of tumorigenesis. However, these events can also occur in the later phases of the disease [53,64]. Indeed, the comparative analysis of cancer cells from primary sites and from metastases revealed an enrichment in chromosomal instability in the metastases of several cancer types [65,66].

These analyses of whole genome data have also demonstrated that metastatic dissemination can be monoclonal or polyclonal. Furthermore, some metastatic clones can have their own subclone evolution through the increase in genome complexity of a metastatic precursor (Figure 4A,B) [67]. The comparative analyses of a metastatic tumor versus a primary tumor have indicated the absence of universal metastatic-specific driver alterations exclusive to metastatic disease. It rather shows a continuous evolution of the tumor with increased genome heterogeneity and complexity [68]. Nevertheless, recent data suggest that certain alterations, also found in primary tumors, are enriched at metastatic sites, revealing the possible existence of drivers specific to metastatic clones (Figure 4A) [68,69]. Moreover, distinct patterns of copy number alterations have been observed in metastases from different tumor types, highlighting that a specific gain/loss of chromosomes confers advantages in certain tumor types [66]. It is still unknown whether these specific drivers confer a better resistance to drugs and/or a better ability to succeed in the metastasis process. Interestingly, the analysis of specific metastatic tropism suggests that some genomic characteristics may be linked with the potential to seed in a specific organ. For example, a gain in semaphorin 4D was shown to support tumor cell transmigration through the blood–brain barrier and MYC has been suggested as a key factor for the adaptation of disseminated tumor cells to the activated brain microenvironment [70].

## 3. Nuclear Squeezing and Its Role in Activating the Innate Immune Response cGAS

**Figure 5:**
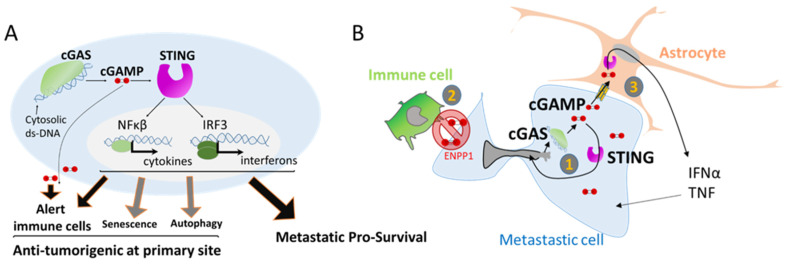
The activation of cGAS can support metastasis survival. (**A**) Activation of inflammatory genes through the detection of double strand DNA (ds-DNA) by the enzyme cGAS. Double strand-DNA bound cGAS induces the production of the second messenger cGAMP that in turn activates STING, leading to the transcription of several inflammatory response genes. cGAMP can also be a paracrine signal by being released in the extracellular compartment or transferred to other cells. cGAS pathway is involved in several processes such as alerting the immune cells but is also involved in senescence, autophagy, and surprisingly in favoring metastasis survival. (**B**) cGAS activation in metastatic cells. (1) cGAMP supports cell own growth as an autocrine signal by the induction of inflammatory genes. (2) To avoid extracellular cGAMP release and activation of immune cell attack, cancer cells express ENPP1 that selectively hydrolyze the extracellular pool of cGAMP. (3) In the context of brain metastasis, cGAMP can transfer to neighbor astrocyte cells by carcinoma–astrocyte gap junctions. This paracrine signal supports the growth of metastatic cells by the astrocytes.

Besides genomic alterations, NER-derived leakage of DNA into the cytoplasm can also trigger an immune response. The presence of DNA in the cytoplasm can be interpreted as a viral or bacterial attack, and mammalian cells have several mechanisms to detect intrusion and trigger an anti-viral immune response. One of these responses is the activation of the cyclic GMP-AMP synthase (cGAS), a cytosolic DNA sensor that binds to cytosolic ds-DNA and catalyzes the synthesis of the second messenger 2′3′-cyclic-GMP-AMP (cGAMP), which in turn activates STING, eventually leading to the production of several inflammatory factors such as type I interferons, interleukins, and the tumor necrosis factor (Figure 5A) [71]. Importantly this pathway is also activated through the release of ds-DNA from replication stress or from mitochondria DNA damage [71,72].

It is noteworthy that the cGAS/STING pathway has important anti-tumorigenic functions, helping in the clearance of genetically unstable cells by alerting the immune cells (Figure 5A). The secretion of type I interferon favors the establishment of an immune infiltration of T cells [73] that participate in the clearance of defective cells. The secretion of cGAMP into the extracellular space is also an important signal for the activation of dendritic cells and enhanced cross-presentation of tumor-associated antigens to CD8 T cells [74] (Figure 5A). Additionally, the cGAS/STING pathway is also involved in two other barriers against oncogenic transformation by the elimination of pre-cancerous cells through autophagy of cells under crisis [75], and in favoring cell senescence, a permanent arrest of the cell cycle [76] (Figure 5A).

However, recent studies have shown that the cGAS/STING pathway can also be kidnaped by tumor cells to favor tumor progression in metastatic sites. The cGAS/STING pathway can have an autocrine effect by inducing a local inflammation that supports metastatic tumor cell growth [65], opposite to its anti-tumorigenic action at tumor primary site (Figure 5B). Using a mouse model, Bakhoum and coworkers showed that highly genetically unstable cancer cells with high chromosomal instability and an activated cGAS/STING pathway are more prone to form metastases than cancer cells with a more stable genome that do not activate the cGAS/STING pathway [65]. Interestingly, the metastases harboring cancer cells with unstable genomes engage a STING-dependent noncanonical activation of NF-κB and inflammatory responses that favor invasion and metastasis [65,77] (Figure 5B).

Thus, it is intriguing to observe that metastatic tumor cells adopt inflammatory signaling and the induction of chronic inflammation while evading the immune attack in the newly seeded site. A recent study combining data from patients and mouse models has demonstrated that the expression of ENPP1 in metastases is a key factor for this outcome [78]. ENPP1 is an enzyme that can hydrolyze the extracellular cGAMP, preventing its transfer from cancer cells to the microenvironment, thus avoiding its transfer to immune cells [78]. ENPP1 activity leads to a reduction in immune cell infiltration at the metastatic site. In clinic, *Enpp1* expression has been associated with reduced lymphocytic infiltration in human cancers in accordance with the role of ENPP1 in escaping the immune system [78] (Figure 5B).

In addition, at the specific metastatic brain niche, cGAMP can act as a paracrine signal between disseminated cancer cells and their environment. In brain metastases, invasive breast and lung cancer cells establish gap junctions with astrocytes allowing cGAMP transfer. In return, astrocytes activate the innate immune response leading to the secretion of factors that support metastatic growth and chemoresistance [79] (Figure 5B). In this particular study, the origin of the cytoplasmic ds-DNA that leads to cGAMP production was not identified, but it is tempting to speculate that NER can be one of the sources. Then, NER associated genomic instability can initiate a paracrine crosstalk, that is often underestimated in the study of metastasis, providing a pro-survival signaling pathway necessary for its growth.

Moreover, the cGAS/STING pathway can also support metastasis by promoting a welcoming tumor microenvironment. cGAS is indispensable for senescence [80] and initiates the secretion of senescence-associated secretory phenotype (SASP) [81]. SASP paracrine signaling from cells that failed to form metastasis can mediate several pro-tumorigenic effects, such as promoting the induction of tumor-associated angiogenesis [76]. Then by inducing senescence in cells failing successful metastasis, the cGAS/STING pathway influences and primes the tumor microenvironment.

To conclude, cGAS/STING pathway activation can have opposite outcomes depending on its location. In primary tumor sites, the cGAS/STING pathway has an anti-tumorigenic action, being a major driver of cancer immunity, while at metastatic sites, this pathway has a pro-survival activity.

## 4. Discussion

The mechanics and physics behind metastasis are frequently underestimated. Studies have been limited to the point of view of the behavior and movement of the cytoskeleton; however, the mechanical cues derived from cell motility can have much more of an impact on tumor progression than anticipated. A better comprehensive approach could highlight the different roles of migration and nuclear squeezing, from their effect on cellular mechanoresponsivity and in epigenetic switch, to their role in genome diversity generation, and finally their implication in inducing the cGAS/STING pathway.

During metastasis, tumor cells need to overcome several barriers in order to access circulation and migrate to new locations. Cancer cells from epithelial origin do not always have intrinsic invasive and migration properties. Thus, the reprogramming of cancer cells is often necessary for a successful metastatic process. The transient embryogenic reprogramming EMT has been associated with the metastatic process, conferring invading and migrating properties [82], notably by inducing changes in cytoskeleton organization. EMT is a highly plastic process [83] that often presents a continuum of hybrid epithelial–mesenchymal states. EMT can be modulated by several factors such as the gradient of several cytokines as TGFβ, a well-known EMT inducer, present under inflammation and in the tumor microenvironment [84]. Another aspect of EMT plasticity can be the mechanical forces. As mentioned before, cells under constriction can sense the constricted area, and undergo nuclear deformation that triggers chromatin loosing [43], and increases the cytoskeleton forces [44,45] to allow a faster escape from the constriction. Those rapid changes in the cytoskeleton can be interpreted as a hybrid E-M state. The mechanosensors from the plasmatic membrane can also sense the matrix stiffness and can modulate the cytoskeleton, as well as the EMT process [37,40]. Altogether, such mechanoresponses favor the diversity in EMT hybrid states due to its rapid changes. The diversity of traction movements due to diverse ECM environments such as pore and channels size and presence of fibers can explain the heterogeneity in EMT states observed in cancer patients [85].

Nuclear squeezing [46,47] and extensive forces during migration [34] can lead to transient NER. Noteworthy, nuclear envelope fragility and NER are also associated to other events [27] such as viral infection [86,87,88,89], some neuropathies [90], aging [91] or as a consequence of impairment in lamin levels [91,92,93] or function as in some type of laminopathy [94]. Over the past 10 years, the research on NE biology has revealed unexpected consequences of its deregulation and has shown the implication of NER in unexpected diseases such as in cancer. From a genomic point of view, NER can lead to an extensive genomic diversity, an ally of tumor progression. Resistance against anticancer therapy is acquired through the development of new mutations, chromosomal reorganization and new copy number variations to find new way to bypass the therapy (Figure 4). Studies from NER of micronuclei and cells with chromatin bridge have shown that NER originates a tremendous variety of genomic reorganizations that are also found during cancer progression (Figure 3). Each cell can gain new genomic aberrances, increasing the pool of cells with unique genomic combination. It is a lottery and hazardous process that might favor or disfavor growth.

Results from clinic support this premise. Technological advances have allowed the isolation and study of CTC from patients, contributing to the detection of subpopulations that comprise intra-tumor heterogeneity. It also permitted the detection of acquired mutations from metastatic sites that could be used as a diagnostic tool toward personalized medicine during the progression of the disease [95,96]. Genomic analysis have shown important heterogeneity between CTC population isolated from the same patient in accordance with the genomic evolution of tumors [96,97,98,99,100,101]. It is likely that the NER that takes place during tumor cell migration is directly involved in the genomic modifications observed in CTC. As human metastases isolated from different sites are enriched in chromosomal instability [65,66], it is tempting to speculate that NER plays a major role in the establishment of genomic diversity during tumor progression.

The field of oncology is taking an increasing interest in understanding the tight connection between immune system and cancer cells. Recently two major innate immune mechanisms have come into the limelight. First APOBEC, a DNA mutator participating in the inhibition of retrovirus and retrotransposon mobility. This enzyme binds preferably to DNA stem-loops [102] and is responsible for one of the most prominent mutation signatures in cancer, present in over half of human tumors, called the APOBEC signature, or Signatures 2/13 and might be also involved in the Aging signature [103]. APOBEC seems also to be responsible of the Kataegis and appears to be linked with nuclear envelope disruption rupture [60,61]. Interestingly, DNA damage and replication stress also seems to stimulate the expression of this enzyme [104]. The second, is the cGAS/STING pathway, a recently discovered pathway [105]. Remarkably, the cGAS/STING pathway, initially involved in tumor cell clearance has also been shown to act as a pro-survival pathway in metastases [65,78,79]. This contradictory action might be a consequence of doses and gradients. In primary tumor sites, levels of extracellular cGAMP and secreted interferon type 1 should be higher than in a metastatic site which only contained few seeded tumor cells. Furthermore, a moderate and transient activation of the innate immune system, as observed during transient NER or due to the limited amount of DNA from micronuclei could be the Achilles heel, by having a protective effect rather than a detrimental one.

Activation of the cGAS/STING pathway leads to the release of several interleukins and interferons in an autocrine or a paracrine fashion. Such signals are indispensable for metastasis survival. Disseminating cells are known to ’talk ‘to the microenvironment and can educate cells around them such as fibroblast (e.g., CAF) to deliver the proper growth factors tailored to their needs. For example, in case of prostate disseminate cells, single cell RNAseq analysis of dormant and proliferative metastatic cells have shown a cross-talk between cancer cells and fibroblasts. Proliferative metastatic cells secrete prostaglandin PGE2 that activates prolactin secretion in nearby fibroblasts, which facilitates tumor cell proliferation [106]. In the case of the cGAS/STING pathway, cGAMP is a signal that induces the secretion of several pro-inflammatory cytokines such as the senescence-associated secretory phenotype (SASP), known to support angiogenesis and tumorigenesis but as well as interferons type 1 that alert the immune system. Such paradox is still under study and highlight the complexity of the crosstalk between cancer cells and their microenvironment. The growing interest in the cGAS/STING pathway is such that several molecules and strategies to target this route are already delineated and evaluated in preclinical models and in clinical trials for autoimmune diseases [107,108]. Because the primary role of cGAS/STING involves tumor clearance, the efforts were focused creating cGAS/STING agonists. However, early results of clinical trials showed limited efficiency [77], leading to a gear switch to antagonist molecules that could give a better result in blocking metastasis survival [77,107]. The surprising link between innate immune system and cancer will reveal new understanding and could lead to new therapeutic targets in the future.

Metastasis is extremely inefficient; it is estimated that less than 1% of cells that intravasate into the blood stream will ultimately succeed in the generation of distant metastasis [7]. This inefficiency maybe be the result of the mechanical stress excess. The extreme nuclear squeezing during ECM migration, insertion into the transendothelial wall or passage through tiny capillaries can lead to massive DNA damage, that could be deadly or conferring unfavorable mutations. Moreover, metastatic cells can also fail to escape the immune system after cGAS activation. However, mechanical forces may be indispensable for a successful metastasis process. Surprisingly, in a study that fluctuates the density of the BM, it was showed that a BM with loosen connections and bigger pores, impaired the success of metastasis [1]. This data was also observed in breast cancer patients in which loosen BM is a good prognostic factor [1]. The mechanism behind this surprising result could be a balance of mechanoresponsivity that prime migration, induction of genomic instability and activation of the cGAS pathway that favors metastasis survival.

## 5. Conclusions

To conclude, mechanical stress and cellular squeezing during the migration, invasion, and circulation of tumor cells have an unsuspected and indispensable role in tumor progression. The recent discovery of NER during the interphase has highlighted its role in the creation of complex chromosomic karyotypes in line with tumor evolution. Moreover, the release of DNA into the cytoplasm activates the innate immune response cGAS/STING pathway that supports metastases growth in different ways. These recent discoveries could be an opportunity to find new therapeutic targets to suppress metastasis, the principal cause of death in cancer patients. The relationship between NE integrity, genome integrity and activation of the immune system still have some secrets to reveal and could be more important than suspected during the development of diseases.
